# Supplementary material for: Epstein–Barr Virus LMP1 Induces Soluble PD-L1 in Nasopharyngeal Carcinoma
Source: Microorganisms. 2021 Mar 15;9(3):603. doi: 10.3390/microorganisms9030603 (PMC7998736; doi:10.3390/microorganisms9030603)
Supplement: Supplementary file 1 [file microorganisms-09-00603-s001.pdf]

# Supplementary files (revised version)
# Epstein–Barr virus LMP1 induces soluble PD-L1 in nasopharyngeal carcinoma

Kina Kase, Satoru Kondo, Naohiro Wakisaka, Hirotomo Dochi, Harue Mizokami, Eiji Kobayashi, Makoto Kano, Takeshi Komori, Nobuyuki Hirai, Takayoshi Ueno, Yosuke Nakanishi, Miyako Hatano, Kazuhira Endo, Makiko Moriyama-Kita, Hisashi Sugimoto, Tomokazu Yoshizaki.

## Supplementary file (revised version).
## Figure S1.

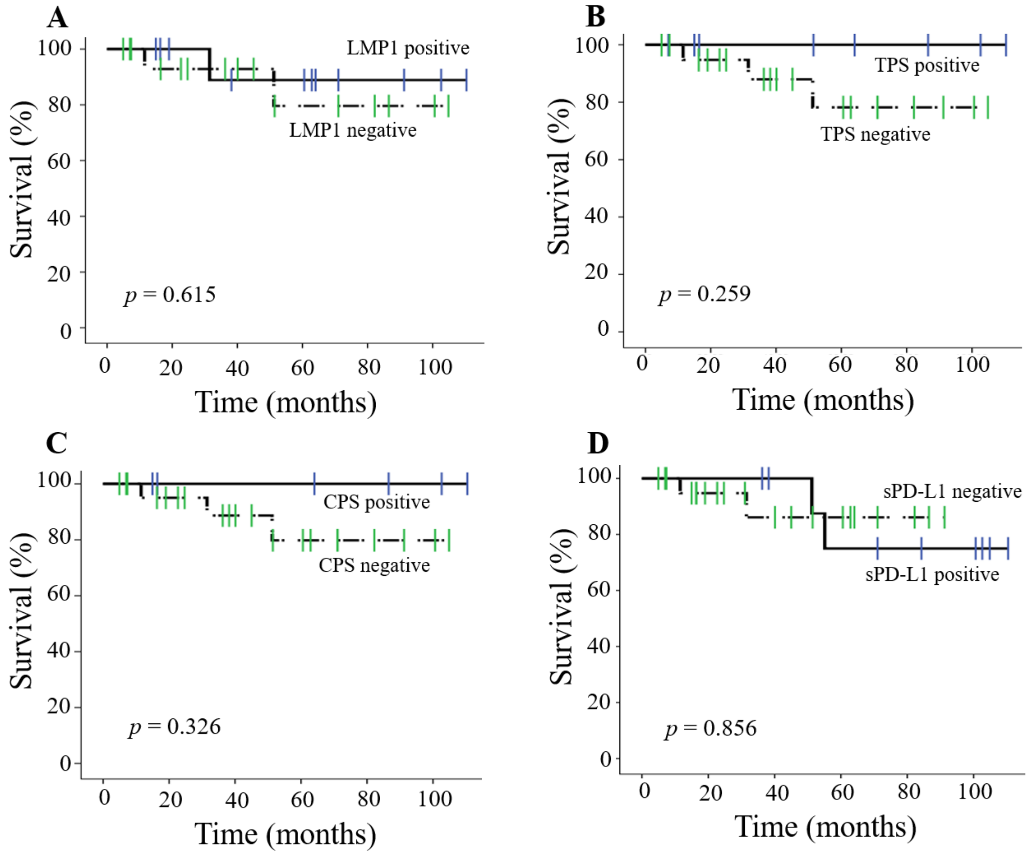


**FigureS1**. Kaplan–Meier curves of overall survival (OS) in patients with NPC. The *p*-value was calculated using log-rank test. (A) Kaplan–Meier curves of LMP1-positive (⩾ LMP1 expression score 10 %) and LMP1-negative (< LMP1 expression score 10 %) samples. (B) Kaplan–Meier curves of PD-L1 TPS-positive (⩾ 10 %) and PD-L1 TPS-negative (< 10 %) samples. (C) Kaplan–Meier curves of PD-L1 CPS-positive (⩾ 10 %) and PD-L1 CPS-negative (< 10 %) samples. (D) Kaplan–Meier curves of sPD-L1-positive (⩾ 0.1 ng/mL) and sPD-L1-negative (< 0.1 ng/mL) samples. $*p < 0.05$. LMP1: latent membrane protein 1, TPS: tumor proportion score, CPS: combined positive score, sPD-L1: soluble PD-L1.

# Supplementary files (revised version)
# Epstein–Barr virus LMP1 induces soluble PD-L1 in nasopharyngeal carcinoma

Kina Kase, Satoru Kondo, Naohiro Wakisaka, Hirotomo Dochi, Harue Mizokami, Eiji Kobayashi, Makoto Kano, Takeshi Komori, Nobuyuki Hirai, Takayoshi Ueno, Yosuke Nakanishi, Miyako Hatano, Kazuhira Endo, Makiko Moriyama-Kita, Hisashi Sugimoto, Tomokazu Yoshizaki.

Supplementary file (revised version).
Table S1.

**Table S1.** Cox proportional hazard regression analysis in the 32 patients with NPC (OS)

| Characteristics | Univariate analysis | | Multivariate analysis | |
|---|---|---|---|---|
| | **Hazard ratio (95%CI)** | ***p* Value** | **Hazard ratio (95%CI)** | ***p* Value** |
| Gender (male) | 16.432 (1.014 – 266.243) | 0.049* | | |
| Smoking (smoking) | 0.262 (0.037 – 1.867) | 0.181 | | |
| Alcohol (drinking) | 0.401 (0.056 – 2.885) | 0.364 | | |
| Tumor (T3-4) | 0.764 (0.107 – 5.469) | 0.789 | | |
| Lymph node (positive) | 25.241 (0 – 25779575.16) | 0.653 | | |
| Metastasis (positive) | 0.043 (0 – 151971.975) | 0.682 | | |
| Stage (III-IV) | 25.122 (0 – 7610851.947) | 0.617 | | |
| PD-L1 TPS (positive) | 35.685 (0.001 – 1262055.418) | 0.504 | | |
| PD-L1 CPS (positive) | 31.326 (0 – 2470875.866) | 0.549 | | |
| LMP1 (positive) | 1.836 (0.166 – 20.265) | 0.62 | | |
| sPD-L1 (⩾0.1ng/mL) | 0.833 (0.115 – 6.017) | 0.856 | | |

*$p < 0.05$. NPC: nasopharyngeal carcinoma, OS: overall survival, CI: confidence interval, LMP1: latent membrane protein 1, TPS: tumor proportion score, CPS: combined positive score, sPD-L1: soluble PD-L1.
